# Loss of maternal EED results in postnatal overgrowth

**DOI:** 10.1186/s13148-018-0526-8

**Published:** 2018-07-13

**Authors:** Lexie Prokopuk, Jessica M. Stringer, Craig R. White, Rolf H. A. M. Vossen, Stefan J. White, Ana S. A. Cohen, William T. Gibson, Patrick S. Western

**Affiliations:** 1 Centre for Reproductive Health, Hudson Institute of Medical Research and Department of Molecular and Translational Science, Monash University, Clayton, Victoria 3168 Australia; 2 Monash Biomedicine Discovery Institute, Monash University, Clayton, Victoria 3800 Australia; 3 Centre for Geometric Biology, School of Biological Sciences, Monash University, Clayton, Victoria 3800 Australia; 4 Leiden Genome Technology Centre, Department of Human Genetics, Leiden University Medical Center, Leiden, the Netherlands; 5 Department of Medical Genetics, University of British Columbia and British Columbia Children’s Hospital Research Institute, Vancouver, BC Canada

**Keywords:** Epigenetic inheritance, Germ, Oocyte, Polycomb, Histone, Weaver, EED, EZH2, Overgrowth, H3K27me3

## Abstract

**Background:**

Investigating how epigenetic information is transmitted through the mammalian germline is the key to understanding how this information impacts on health and disease susceptibility in offspring. EED is essential for regulating the repressive histone modification, histone 3 lysine 27 tri-methylation (H3K27me3) at many developmental genes.

**Results:**

In this study, we used oocyte-specific *Zp3-Cre recombinase (Zp3Cre)* to delete *Eed* specifically in mouse growing oocytes, permitting the study of EED function in oocytes and the impact of depleting EED in oocytes on outcomes in offspring. As EED deletion occurred only in growing oocytes and females were mated to normal wild type males, this model allowed the study of oocyte programming without confounding factors such as altered in utero environment. Loss of EED from growing oocytes resulted in a significant overgrowth phenotype that persisted into adult life. Significantly, this involved increased adiposity (total fat) and bone mineral density in offspring. Similar overgrowth occurs in humans with Cohen-Gibson (OMIM 617561) and Weaver (OMIM 277590) syndromes, that result from de novo germline mutations in *EED* or its co-factor *EZH2*, respectively. Consistent with a role for EZH2 in human oocytes, we demonstrate that de novo germline mutations in *EZH2* occurred in the maternal germline in some cases of Weaver syndrome. However, deletion of *Ezh2* in mouse oocytes resulted in a distinct phenotype compared to that resulting from oocyte-specific deletion of *Eed*.

**Conclusions:**

This study provides novel evidence that altering EED-dependent oocyte programming leads to compromised offspring growth and development in the next generation.

**Electronic supplementary material:**

The online version of this article (10.1186/s13148-018-0526-8) contains supplementary material, which is available to authorized users.

## Background

Factors regulating oocyte (egg) and sperm programming and early embryonic development have been associated with the fetal origins of disease, including reduced cognitive ability and increased chronic diseases, such as type-2 diabetes, obesity, heart disease and behavioural anomalies [1–5]. The causes of these defects are poorly understood, but are likely to be in part due to altered epigenetic programming of oocytes or sperm that significantly impact on embryonic development and underlie the fetal origin of some of these disorders [1, 2, 4]. Defining how inherited epigenetic information regulates fetal development and postnatal phenotypic outcomes is therefore important for understanding how inherited epigenetic information impacts on human health and disease. While the establishment of aberrant epigenetic states has been associated with disease, functional analyses of epigenetic inheritance that isolate in vivo effects generated in the germline are not possible in humans. Here, we have used genetic mouse models to mediate oocyte-specific deletion of *Eed* and *Ezh2* to understand how changes in epigenetic information established in the oocyte can impact on phenotypic outcomes in offspring.

H3K27me3 is a critical epigenetic modification that is catalysed by polycomb repressive complex 2 (PRC2), a highly conserved epigenetic modifying complex. PRC2 is comprised of three core protein subunits: Embryonic Ectoderm Development (EED), Enhancer of Zeste 1/2 (EZH1/2) and Suppressor of Zeste 12 (SUZ12). Global loss of function to any one of these components results in drastically compromised enzymatic activity of PRC2, substantial loss of H3K27me3 and embryonic lethality in mice [6–8]. Recent studies of germ cells have demonstrated enrichment of H3K27me3 at key developmental genes in male and female germ cells [9–11] and the maintenance of some H3K27me3 marked histones in mature sperm [12–14]. Furthermore, oocyte-specific deletion of *Ezh2* results in loss of H3K27me3 in the zygote and ~ 40% growth restriction in maternal offspring [15]. In humans, de novo germline mutations in *EED* or *EZH2* lead to Cohen-Gibson (OMIM 617561) or Weaver (OMIM 28229590) syndromes, which are characterised by overgrowth, skeletal defects and advanced bone age [16–23], indicating that PRC2 activity may be required in the human germline for regulating outcomes in offspring. Together, these studies raise the possibility that PRC2 and H3K27me3 may underpin epigenetic inheritance effects on offspring development and postnatal outcomes.

During female fetal development, germ cells commit to oogenesis and enter meiotic prophase [24]. Folliculogenesis occurs in early postnatal life and results in establishment of a finite pool of quiescent primordial follicles that underpin the female reproductive lifespan. Subsequently, primordial follicles are continuously released in reproductively mature females, initiating a prolonged period of oocyte growth and maturation that takes around 21 days in mice and up to 12 months in humans [25]. While most of this period involves oocyte growth while in a diploid state, it is completed by rapid maturation of each oocyte through meiosis I and meiosis II, ultimately producing a haploid oocyte at fertilisation [26]. Before completion of the first meiotic division, the diploid oocyte enters the germinal vesicle (GV) stage, characterised by decondensed chromatin and a period of high transcription that includes the production of maternal factors (proteins and RNAs) that are required for directing preimplantation development in the offspring [27]. In addition to maternal factors, the mature oocyte carries specific epigenetic information required for offspring development, but the nature, extent and effects of this information on offspring development are not yet fully understood [28, 29].

Germ cell-specific knockout mouse models have been valuable for analysing the function of maternal factors in the absence of confounding effects such as in utero environment and nutritional influences derived from the mother [15, 28, 30]. *Zp3Cre*, is a well-established model for generating oocyte-specific gene deletion mediated by transcription of *Cre recombinase* under control of the *Zona Pellucida 3* (*Zp3*) promoter [31]. This model allows the production of offspring from oocytes that lack specific genes only during their maturation and can be effectively used for functional analyses of maternal inheritance [15, 31–33].

Although deletion of the PRC2 component, *Ezh2*, in the growing oocyte leads to restricted growth in offspring [15], little is known about the role of *Eed* in the female germline. However, the PRC1 components, *Ring finger protein 2* (*Rnf2*) and *Ring finger protein 1* (*Ring1*), are regulated throughout oogenesis and absence of maternal RNF2 and RING1 proteins leads to developmental arrest at the two-cell stage of embryogenesis [30]. Moreover, injection of the H3K27me3-specific demethylase, *Kdm6b*, into zygotes resulted in ectopic maternal expression of specific genes that are normally expressed only from the paternal allele, demonstrating that maternal H3K27me3 regulates DNA methylation-independent imprinting [34]. These data suggest oocyte-specific roles for polycomb group proteins in the establishment of maternal factors and/or epigenetic modifications that are required for correct development of offspring.

In this study, we used *Zp3Cre* to delete *Eed* specifically from growing oocytes. The resulting oocytes lacked H3K27me3 and produced offspring with significant overgrowth that involved increased adiposity (percentage fat) and increased bone mineral density. These data demonstrate that EED is required in the oocyte for programming developmental outcomes that affect life-long outcomes in offspring. This contrasted with growth restriction that resulted from deletion of *Ezh2* in growing oocytes, indicating that while EED mediates epigenetic inheritance through the maternal germline, the function of EZH2 is more complex.

## Results

**Fig. 1:**
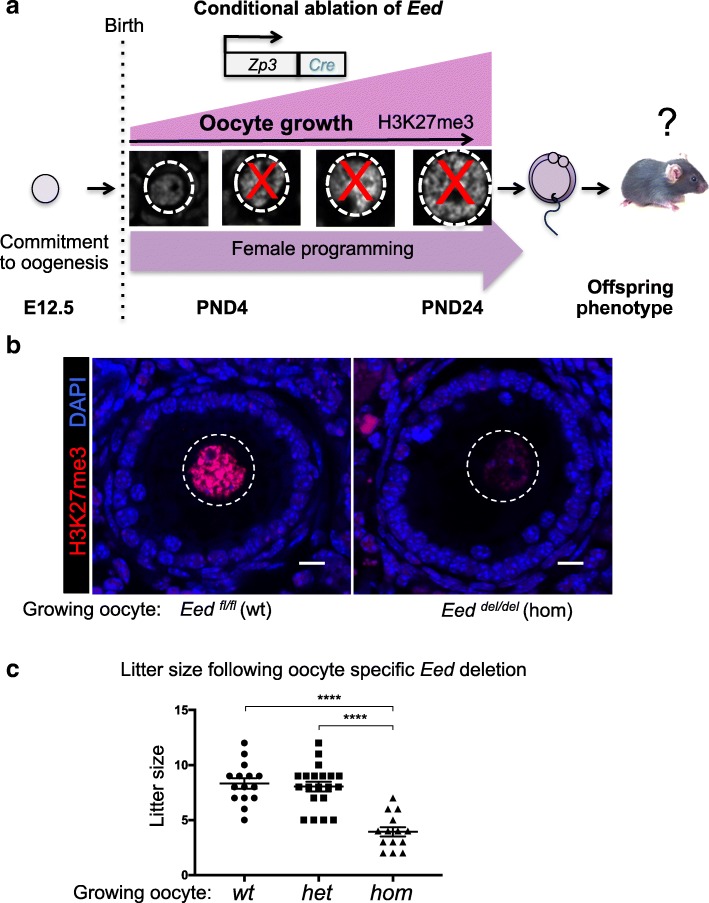
Deletion of *Eed* significantly reduced H3K27me3 in growing oocytes: **a** Schematic of the study aims—germ cells commit to female development after E12.5 and new epigenetic information is established in growing oocytes after birth. H3K27me3 is enriched as oocytes grow, with strong enrichment in the maturing oocyte. Using an *Eedfl-Zp3*Cre mouse model, we investigated the impacts of deleting *Eed* in the growing oocyte on offspring weight and growth. As EED deletion occurs only in growing oocytes, this model allows the study of EED-dependent maternal programming without contributions from confounding factors such as in utero environment. **b** Representative confocal images of immunofluorescence in ovary sections from adult *Eed*^*fl/fl*^ and *Eed*^*fl/fl*^;*Zp3-*Cre female mice producing *Eed*^*fl/fl*^ (wild type; *wt*) and *Eed*^*del/del*^ (homozygous; *hom*) oocytes, respectively. Merged channels: H3K27me3 (red) and DAPI (DNA; blue). *Eed*^*fl/fl*^ (*wt*) and *Eed*^*del/del*^(*hom*) oocytes are shown within the white dashed line. Images are representative of four biological replicates. 10-μm scale bars. **c** Average litter sizes from mothers producing *Eed*^*fl/fl*^ (*wt*), *Eed*^*wt/del*^ (heterozygous; *het*) and *Eed*^*del/del*^ (*hom*) growing oocytes: *n* = 15, 20 and 13 litters per genotype, respectively, and 7 different mothers per genotype group. ****P < 0.0001. One-way ANOVA plus post hoc Tukey’s multiple comparisons test. Error bars ± SEM

Immunofluorescent studies of oocyte development demonstrated that H3K27me3 is increasingly enriched in the nucleus of oocytes during follicle growth [35]. In this study, we used a *Zp3Cre* transgene to mediate oocyte-specific deletion of *Eed*, in order to determine the effect loss of maternal PRC2 function had on offspring (Fig. 1a). The *Eed*^*fl/fl*^ model was originally developed by Orkin and colleagues, and results in deletion of exons 3–6 of *Eed* and loss of the ability for PRC2 to catalyse H3K27 methylation [36]. Genotyping of progeny from *Eed* floxed females carrying *Zp3Cre* mated to wild type males demonstrated that *Eed* deletion was 100% efficient in oocytes (Additional file 1: Figure S1; *Chi-square test*, *nsd*). Mouse oocyte growth encompasses 22–24 days, after which they complete meiosis during maturation within a single day, ultimately producing haploid ova [37, 38]. Deletion using *Zp3Cre* occurs early during oocyte growth, resulting in loss of the target gene during the majority of the diploid (4C) oocyte growth phase [31]. Critically, immunofluorescence demonstrated that *Eed*^*del/del*^ oocytes contained markedly reduced nuclear H3K27me3, consistent with substantial loss of PRC2 function in these cells (Fig. 1b). Initially, we determined whether deleting *Eed* in oocytes affected average litter size produced from *Eed*^*wt/wt*^ *Eed*^*wt/del*^ and *Eed*^*del/del*^ growing oocytes. Despite severe depletion of H3K27me3, *Eed*^*del/del*^ oocytes produced live born pups in normal sex ratios. However, the average litter size produced by females producing *Eed*^*del/del*^ oocytes was significantly reduced compared to females producing *Eed*^*wt/wt*^ or *Eed*^*wt/del*^ oocytes, although the causes for the litter size reduction remain unknown (Fig. 1c, *n* = 13–20 litters per genotype). This model therefore provides an opportunity to study offspring derived from oocytes that lack EED and H3K27me3, specifically during oocyte growth, maturation and preimplantation development, prior to activation of the paternal *Eed* allele.

**Fig. 2:**
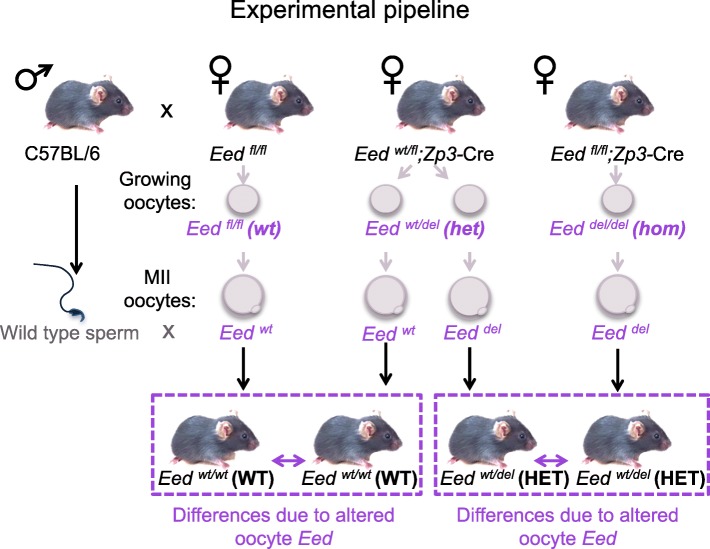
Schematic of experimental breeding to determine epigenetic differences in isogenic offspring from *Eed floxed* females: Wild type males were mated with *Eed*^*fl/fl*^, *Eed*^*fl/del*^;*Zp3-*Cre and *Eed*^*fl/del*^;*Zp3Cre* female mice that produce *Eed*^*del*^ *or Eed*^*wt*^ haploid oocytes derived from *Eed*^*fl/fl*^ (*wild type*; *wt*), *Eed*^*wt/del*^(*heterozygous*; *het*) or *Eed*^*del/del*^ (*homozygous*; *hom*) growing oocytes. *Het* oocytes grow and mature in the presence of functional EED, while *hom* oocytes grow and mature in the absence of EED function. Production of offspring by mating *Eed*^*fl/del*^;*Zp3-*Cre and *Eed*^*fl/del*^;*Zp3Cre* female mice with isogenic wild type males allowed the comparison of isogenic HET offspring from *het* and *hom* oocytes. As the resulting HET offspring were isogenic and carry identical heterozygous *Eed* deletion, differences detected could be ascribed to loss of epigenetic regulation by EED in the oocyte and before cavitation of paternal *Eed* in the preimplantation embryo. Similar comparisons were made between WT offspring produced from *Eed*^*fl/fl*^ and *Eed*^*fl/del*^;*Zp3-*Cre females*.* Comparison of WT and HET offspring produced by *Eed*^*fl/fl*^ and *Eed*^*fl/del*^;*Zp3-*Cre females provides an internal control identifying the contribution of *Eed* heterozygosity to the phenotype. Genetically identical offspring are shown in purple dashed box

Mating females with oocyte-specific deletion of *Eed* to wild type C57BL/6 males provided the opportunity to compare isogenic offspring in the absence of confounding maternal in utero effects (Fig. 2). Importantly, oocytes developing in *Eed*^*wt/fl*^;*Zp3-Cre* transgenic mothers have one intact copy of *Eed* during the oocyte growth period, when epigenetic modifications including DNA methylation and H3K27me3 are established. In contrast, oocytes from *Eed*^*fl/fl*^;*Zp3-Cre* transgenic females lack both copies of *Eed* (*Eed*^*del/del*^) and have no EED function in their growing oocytes. Therefore, in this model, we expect the epigenome of *Eed*^*del*^ haploid oocytes produced by *Eed*^*fl/wt*^;*Zp3-Cre* females to be relatively normal as they developed in the presence of reduced, yet sufficient, EED function during their growth and maturation. However, *Eed*^*del*^ haploid oocytes produced by *Eed*^*fl/fl*^;*Zp3-Cre* females lacked EED protein during oocyte growth and maturation (Fig. 2).

This model allowed the production of heterozygous (HET) offspring from females producing *Eed*^*del/del*^ (*hom*) or *Eed*^*wt/del*^ (*het*) growing oocytes mated to wild type males. Although HET offspring from *Eed*^*del/del*^ or *Eed*^*wt/del*^ growing oocytes were isogenic, they have two critical differences. Firstly, we expect these offspring to be epigenetically different, reflecting the presence or absence of EED function in the growing oocyte. Secondly, these offspring lacked maternal EED during the zygotic to paternal genome activation phase of preimplantation development. Therefore, we proposed that differences between the HET offspring from females producing *Eed*^*del/del*^ or *Eed*^*wt/del*^ growing oocytes could be attributed to altered programming in *Eed*^*del/del*^ growing oocytes and during the earliest stages of preimplantation development. Similar comparisons were made between wild type (WT) offspring from *Eed*^*wt/fl*^;*Zp3*-Cre and *Eed*^*wt/wt*^ females, which respectively produced *Eed*^*wt/del*^ and *Eed*^*wt/wt*^ growing oocytes (Fig. 2).

**Fig. 3:**
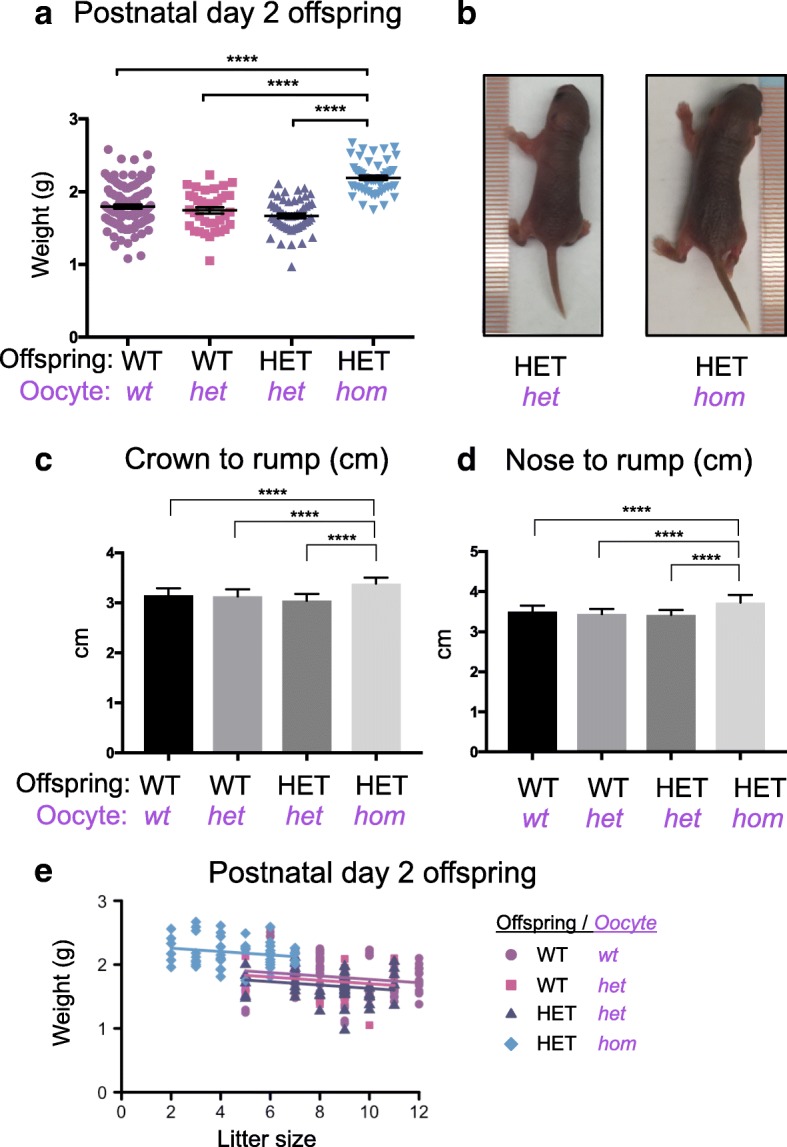
Offspring from *Eed*^*del/del*^ oocytes have increased weight and length that is independent of litter size. **a** Postnatal day (PND) 2 weights of WT and HET offspring produced from *Eed*^*fl/fl*^ (*wt*), *Eed*^*wt/del*^ (*het*) and *Eed*^*del/del*^(*hom*) oocytes and wild type sperm (WT offspring from *wt* oocytes *n* = 118; WT offspring from *het* oocytes *n* = 37; HET offspring from *het* oocytes *n* = 52; HET offspring from *hom* oocytes *n* = 51). **b** Representative images showing two isogenic PND2 male pups. Left: HET offspring from a *het* growing oocyte; Right: HET offspring from a *hom* growing oocyte. **c** Crown to rump measurements of PND2 male and female pups. **d** Nose to rump measurements of PND2 male and female pups. **c**–**d** WT offspring from *wt* oocytes *n* = 29; WT offspring from *het* oocytes *n* = 10; HET offspring from *het* oocytes *n* = 10; HET offspring from *hom* oocytes *n* = 23) ****P < 0.0001. One-way ANOVA plus post hoc Tukey’s multiple comparisons test. Data represents mean ± SEM. **e** Relationship between PND2 weight and litter size: Litter size vs offspring weight (*t*_33.5_ = − 1.32, *P* = 0.20; variance components: litter ID = 0.0398, residual = 0.0345). Accounting for litter size vs offspring weight: HET pups from *Eed*^*del/del*^ oocytes were heavier than HET pups produced from *Eed*^*wt/del*^ oocytes (*t*_34.8_ = 3.44, *P* = 0.002), WT pups from either *Eed*^*wt/del*^ (*t*_36.3_ = 2.81, *P* = 0.008) and *Eed* ^*wt/wt*^ (*t*_33.1_ = 2.29, *P* = 0.03) oocytes

To identify postnatal differences in offspring due to compromised EED-dependent programming in the oocyte and early development, we initially weighed postnatal day (PND) 2 pups generated from *Eed*^*del/del*^, *Eed*^*wt/del*^ and *Eed*^*wt/wt*^ growing oocytes and isogenic wild type sperm (Figs. 2 and 3a). Remarkably, complete loss of EED specifically in the growing oocyte resulted in a 29% increase in weight of PND2 HET offspring from *Eed*^*del/del*^ growing oocytes (average weight 2.19 g, *n* = 51) compared to PND2 HET offspring from *Eed*^*wt/del*^ growing oocytes (average weight 1.69 g, *n* = 52) (Fig. 3a *P* < 0.0001, one-way ANOVA). In addition, HET offspring from *Eed*^*del/del*^ growing oocytes had increased nose to rump length, compared to isogenic age-matched HET offspring from *Eed*^*wt/del*^ growing oocytes (Fig. 3b–d). Similarly, HET offspring from *Eed*^*del/del*^ growing oocytes were significantly longer and heavier than WT offspring from *Eed*^*wt/del*^ growing oocytes, but there was no difference in length or weight between HET and WT offspring generated from *Eed*^*wt/del*^ or *Eed*^*wt/wt*^ growing oocytes (Fig. 3d). There was no bias in this PND2 overgrowth phenotype between the sexes (Additional file 1: Figure S2).

One possible explanation for the increased pup weight at PND2 was the reduced average litter size observed for females producing *Eed*^*del/del*^ growing oocytes (Fig. 1c). To account for this, we analysed the weight data using linear mixed models to statistically evaluate the litter size dependent, and litter size independent contributions to offspring weight. To account for non-independence of pups from individual litters, we included litter identification as a random effect in the analysis. Initially, we ensured that there were no differences in offspring weight due to litter size differences that were attributable individual oocyte genotypes. This revealed that the relationship between litter size and offspring weight among WT and HET pups produced from *Eed*^*wt/wt*^, *Eed*^*wt/del*^ and *Eed*^*del/del*^ oocytes was consistent (litter size by offspring group interaction: *F*_3,48.4_ = 0.048, *P* = 0.99; variance components: litter ID = 0.0423, residual = 0.0346). Next, we determined the relationship between litter size and offspring weight for all genotypes. This revealed that as litter size decreased, there was a minor increase in offspring weight (*t*_33.5_ = − 1.32, *P* = 0.20; variance components: litter ID = 0.0398, residual = 0.0345; Fig. 3e). However, this effect was not significant and was insufficient to account for the substantial increase in the weight of HET offspring generated from *Eed*^*del/del*^ growing oocytes compared to HET or WT offspring generated from *Eed*^*wt/del*^ or *Eed*^*wt/wt*^ oocytes. In contrast, after removing the weight change attributable to litter size, there remained a highly significant increase in weight of HET pups produced from *Eed*^*del/del*^ growing oocytes compared to HET pups produced from *Eed*^*wt/del*^ oocytes (*t*_34.8_ = 3.44, *P* = 0.002) and WT pups produced from either *Eed*^*wt/del*^ (*t*_36.3_ = 2.81, *P* = 0.008) or *Eed*^*wt/wt*^ (*t*_33.1_ = 2.29, *P* = 0.03) growing oocytes (Fig. 3e). Given that this increase in offspring weight only occurred in the HET pups generated from *Eed*^*del/del*^ growing oocytes and was not accounted for by litter size differences, we concluded that loss of EED in the oocyte and early embryo resulted in substantial, postnatal overgrowth.

As the *Eed* HET pups produced were isogenic, the simplest explanation for this difference is that loss of maternal EED in the mouse oocyte and zygote led to a significant early developmental programming effect that impacted on postnatal weight in offspring from *Eed*^*del/del*^ compared to *Eed*^*wt/del*^ growing oocytes. As EED is known only to regulate epigenetic outcomes, this postnatal effect is likely to result from altered epigenetic programming in the oocyte and early embryo.

**Fig. 4:**
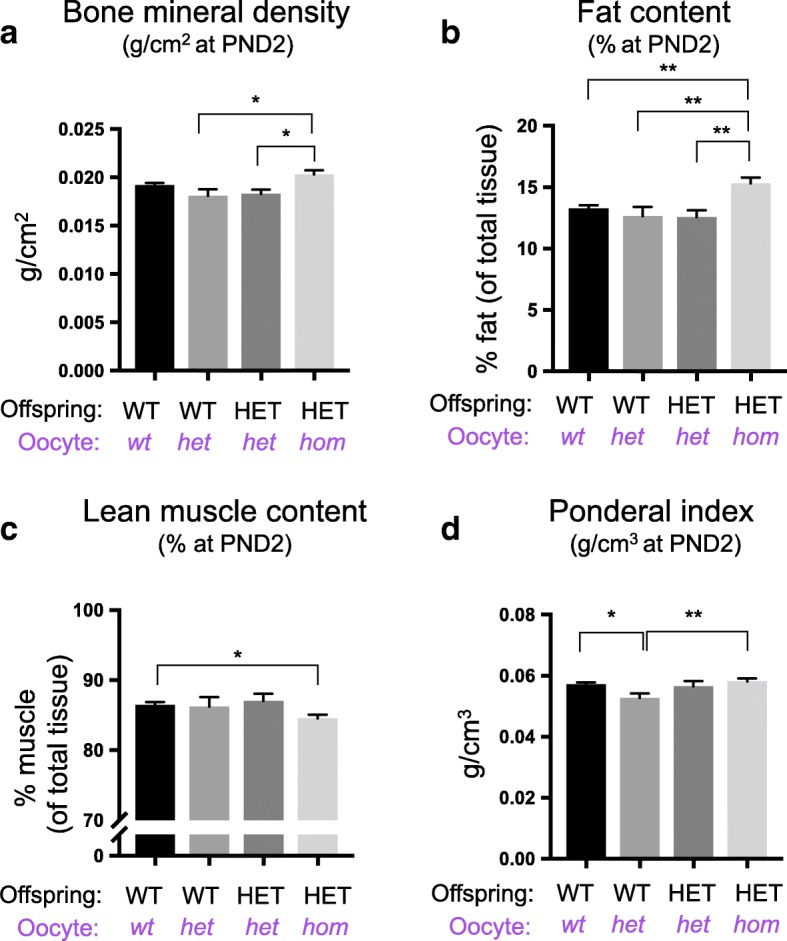
Offspring from *Eed*^*del/del*^ oocytes have increased bone mineral density and fat content, but reduced lean muscle. **a**–**d** Postnatal day (PND) 2: bone mineral density (**a**), lean muscle content (**b**), fat content (**c**), ponderal index (**d**) in WT and HET offspring produced from *Eed*^*fl/fl*^ (*wt*), *Eed*^*wt/del*^ (*het*) and *Eed*^*del/del*^(*hom*) oocytes and wild type sperm (**a**–**d**: WT offspring from *wt* oocytes *n* = 28; WT offspring from *het* oocytes *n* = 6; HET offspring from *het* oocytes *n* = 8; HET offspring from *hom* oocytes *n* = 19). *P < 0.05, **P < 0.01, one-way ANOVA plus post hoc Tukey’s multiple comparisons test. Error bars ± SEM

Recent studies in humans have demonstrated that de novo germline mutations in *EED* lead to Cohen-Gibson (OMIM 617561) syndrome, which is characterised by overgrowth, skeletal defects and advanced bone age [16–20]. DEXA scanning of the PND2 mouse offspring produced in this study revealed increased bone mineral density (BMD) in HET offspring from *Eed*^*del/del*^ growing oocytes compared to HET controls generated from *Eed*^*wt/del*^ growing oocytes (*Eed*^*wt/wt*^ *n* = 28, *Eed*^*wt/del*^ *n* = 14, *Eed*^*del/del*^ *n* = 19; *P* < 0.05, one-way ANOVA; Fig. 4a). Moreover, fat content was also significantly increased in PND2 HET offspring from *Eed*^*del/del*^ oocytes compared to HET offspring from *Eed*^*wt/del*^ growing oocytes, and WT offspring from *Eed*^*wt/del*^ and *Eed*^*wt/wt*^ oocytes (Fig. 4b, *P* < 0.01, one-way ANOVA). Lean muscle content was significantly reduced in HET offspring from *Eed*^*del/del*^ growing oocytes, compared to WT offspring from *Eed*^*wt/wt*^ oocytes (Fig. 4c, *P* < 0.05, one-way ANOVA), but there was no significant difference between HET offspring from *Eed*^*wt/del*^ and *Eed*^*del/del*^ oocytes. The ponderal index (weight/crown-length^3^) of WT and HET offspring from *Eed*^*wt/wt*^ and *Eed*^*wt/del*^ growing oocytes, respectively, was not significantly different indicating that there was no substantial effect on corpulence in these offspring. However, while there was an unexpected marginal decrease in ponderal index in WT pups from *Eed*^*wt/del*^ growing oocytes compared to all other treatment groups, the significance of this change remains unknown (Fig. 4d, *P < 0.05, **P < 0.01 one-way ANOVA).

**Fig. 5:**
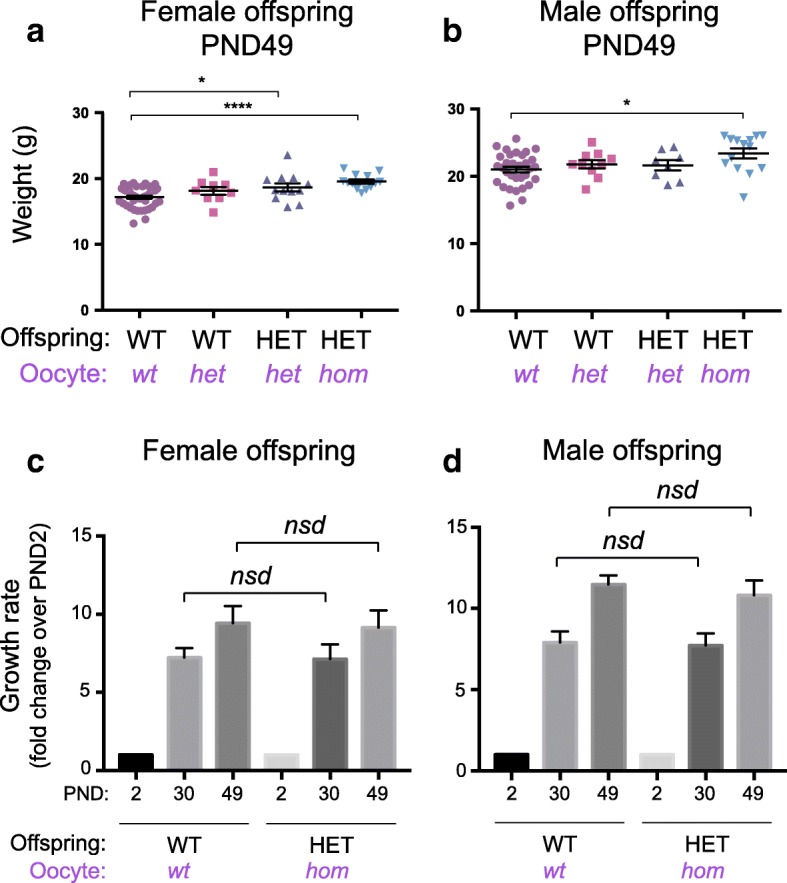
Offspring from *Eed*^*del/del*^ oocytes have increased weight into adulthood. **a**–**b** Postnatal day (PND) 49 weights of female (**a**) and male (**b**) WT and HET offspring produced from *Eed*^*fl/fl*^ (*wt*), *Eed*^*wt/del*^ (*het*) and *Eed*^*del/del*^(*hom*) oocytes and wild type sperm (WT offspring from *wt* oocytes **a**: *n* = 43, **b** 32; WT offspring from *het* oocytes **a** *n* = 9, **b** *n* = 10; HET offspring from *het* oocytes **a** *n* = 12, *b n* = 8; HET offspring from *hom* oocytes **a** *n* = 13, *b n* = 14). **c** Average growth trajectories calculated from average weights of female WT and HET offspring at PND2, 30 and 49 (WT offspring from *wt* oocytes PND2: *n* = 14, PND30: *n* = 14, PND49: *n* = 14; HET offspring from *hom* oocytes PND2: *n* = 10, PND30: *n* = 10, PND49: n = 10). **d** Average growth trajectories calculated from average weights of male WT and HET offspring at PND2, 30 and 49 (WT offspring from *wt* oocytes PND2: *n* = 12, PND30: *n* = 12, PND49: *n* = 12; HET offspring from *hom* oocytes PND2: *n* = 6, PND30: *n* = 6, PND49: *n* = 6). *P < 0.05, ****P < 0.0001; *nsd* represents no significant difference. One-way ANOVA plus post hoc Tukey’s multiple comparisons test. Error bars ± SEM

To determine the weight phenotype observed in PND2 offspring persisted through adult life, female and male offspring from *Eed*^*wt/wt*^, *Eed*^*wt/del*^ and *Eed* ^*del/del*^ growing oocytes were weighed at PND30, PND49 and PND130 (Fig. 5a–b, Additional file 1: Figure S2). HET offspring from *Eed*^*del/del*^ growing oocytes remained significantly heavier than age-matched WT offspring from *Eed*^*wt/wt*^ oocytes at PND30 (females), PND49 and PND130 (Additional file 1: Figure S2). However, there was no statistical difference in male weights at PND30, a growth period encompassing the transition of adolescence to adulthood in mice. Although the reason for this discrepancy remains unknown, individual variation in growth and maturation at this time point may explain the greater individual variability evident in the data. Notwithstanding this limitation, our findings are consistent with Cohen-Gibson patients [16], as the weight differences between animals were partially ameliorated through time and between sexes such that there was no longer a consistent statistically significant difference between HET offspring from *Eed*^*wt/del*^ and *Eed* ^*del/del*^ growing oocytes at PND30 and at older ages (Fig. 5a–b, Additional file 1: Figure S2). Calculation of the change in average sex-specific weights in each group at PND30 and PND49 relative to PND2, detected no difference in the growth rates of WT and HET offspring produced from *Eed* ^*wt/wt*^ and *Eed* ^*del/del*^ growing oocytes (Fig. 5c–d).

**Fig. 6:**
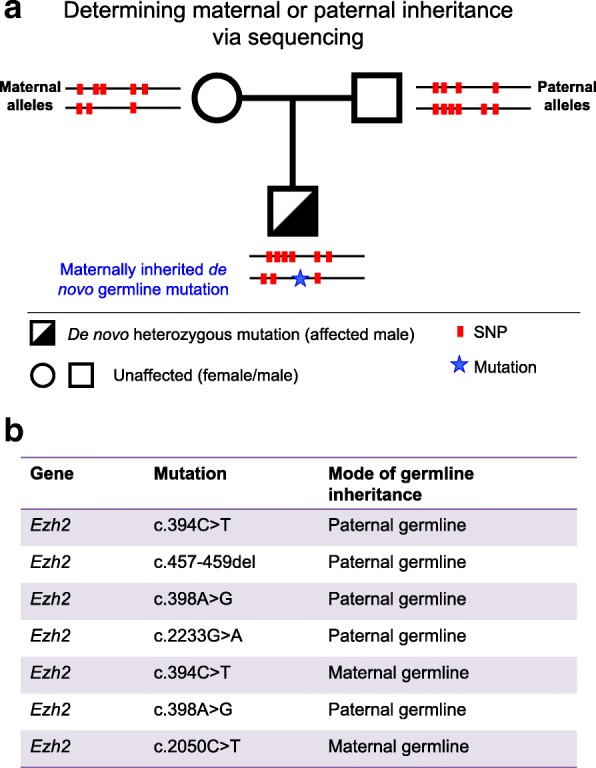
De novo missense mutations in EZH2 are maternally or paternally inherited through the germline in Weaver patients: **a** PacBio sequencing was carried out to identify single nucleotide polymorphisms (SNPs) in DNA from each patient and their respective parental haplotypes. Informative SNPs (i.e., those that were specific to either parent) allowed linkage of the patient’s EZH2 mutation in the patient to either the maternal or paternal allele (example of experimental pipeline shown). **b** The *EZH2* mutation detected in each patient is shown in the middle column and parent-of-origin shown on the right, based on genetic linkage to either the mother or the father

In humans, de novo germline mutations in *EZH2* lead to Weaver Syndrome (OMIM 28229590) and de novo germline mutations in *EED* lead to Cohen-Gibson syndrome, both of which are characterised by overgrowth [16–18, 21–23]. To determine the germline origin of the EZH2/EED de novo mutation, comparative SNPs were analysed in the *EZH2* and *EED* genomic regions in DNA samples from Weaver syndrome patients and their parents (Fig. 6a). Long range, targeted sequencing revealed that the causative EZH2 mutations in human patients occurred either in the maternal or paternal germlines (Fig. 6b). Although patient numbers are quite restricted in this study, parent-of-origin appeared to have no effect on birth weight in human patients Unfortunately, analysis of two patients carrying EED mutations were uninformative, one patient lacked informative SNPs in the sequenced region and data from the other patient was inconclusive as amplification of the template failed for this individual.

**Fig. 7:**
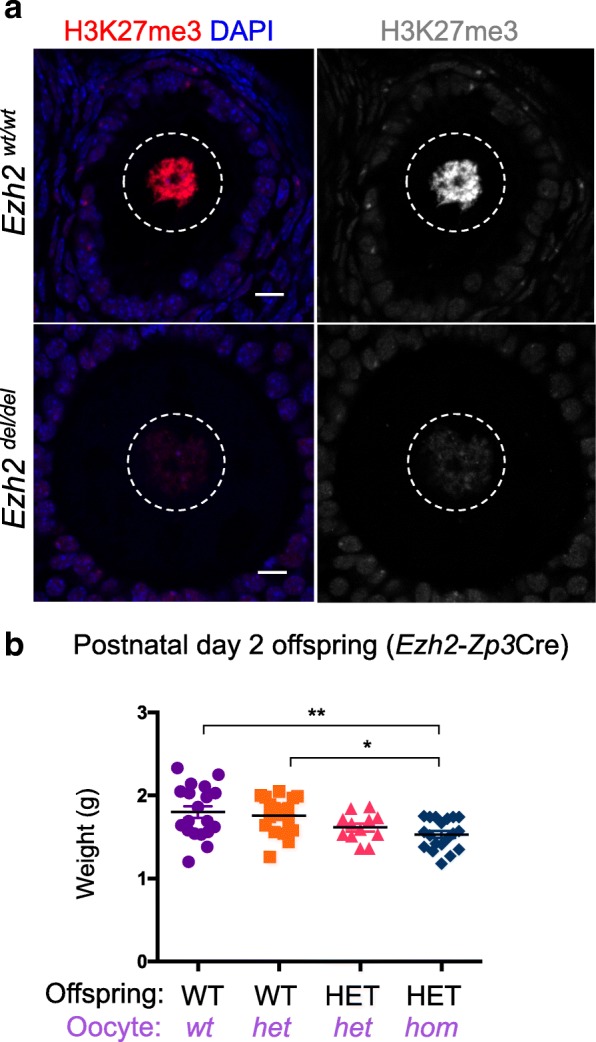
Deletion of *Ezh2* significantly reduced H3K27me3 in growing oocytes and weight in offspring: **a** representative confocal images of immunofluorescence in ovary sections from adult *Ezh2*^*fl/fl*^ and *Ezh2*^*fl/fl*^;*Zp3-*Cre female mice. Left panel—merged H3K27me3 (red) and DAPI (DNA; blue); right panel H3K27me3 shown in greyscale. Oocyte nuclei are shown within the white dashed line. Images are representative of three biological replicates; 10 μm scale bars. **b** Postnatal day (PND) 2 weights of WT and HET offspring produced from *Ezh2*^*fl/fl*^ (*wt*), *Ezh2*^*wt/del*^ (*het*) and *Ezh2*^*del/del*^(*hom*) oocytes and wild type sperm (WT offspring from *wt* oocytes *n* = 19; WT offspring from *het* oocytes *n* = 18; HET offspring from *het* oocytes *n* = 12; HET offspring from *hom* oocytes *n* = 19). *P < 0.05, **P < 0.01, one-way ANOVA plus post hoc Tukey’s multiple comparisons test. Error bars ± SEM

An earlier study in mice found that deletion of *Ezh2* in growing oocytes resulted in reduced birth weight in maternal offspring [15]. As the offspring produced after deletion of *Eed* in growing oocytes resulted in overgrown offspring, we independently generated offspring from *Ezh*^*fl/fl*^ (*Zp3-Cre* negative), *Ezh2*^*wt/fl*^;*Zp3-*Cre and *Ezh2*^*fl/fl*^;*Zp3-*Cre mothers. Consistent with deletion of *Eed* in growing oocytes, deletion of *Ezh2* using the same *Zp3Cre* strategy was highly efficient (Additional file 1: Figure S3) and resulted in substantially reduced H3K27me3 in growing oocytes. The degree to which H3K27me3 was lost in *Ezh2*^*del/del*^ and *Eed*^*del/del*^ oocytes was comparable, indicating that loss of EZH2 is unlikely to be compensated for by the related protein EZH1. In contrast to *Eed*, oocyte-specific deletion of *Ezh2* resulted in significantly reduced PND2 weight in HET offspring from *Ezh2*^*del/del*^ growing oocytes compared to WT pups from *Ezh2*^*wt/del*^ and *Ezh2*^*wt/wt*^ growing oocytes. However, there was no difference in PND2 weight between HET offspring from *Ezh2*^*del/del*^ and *Ezh2*^*wt/del*^ growing oocytes (Fig. 7). Significantly, this indicated that deletion of *Ezh2* in growing oocytes resulted in a genetically defined, heterozygous vs wild type reduction in offspring weight, rather than the epigenetically defined, heterozygous vs heterozygous difference in offspring weight observed for deletion of *Eed* in growing oocytes. A caveat to this is that *Ezh2* HET offspring from *Ezh2*^*wt/del*^ growing oocytes were not significantly different in weight to WT offspring from *Ezh2*^*wt/del*^ or *Ezh2*^*wt/wt*^ growing oocytes. Therefore, it is likely that this phenotype results from a combination of genetic and epigenetic effects mediated through the maternal germline.

## Discussion

We have established a model in which oocyte-specific deletion of *Eed* results in overgrowth in postnatal offspring. Heterozygous offspring from *Eed*^*del/del*^ growing oocytes had increased body weight, length, fat content and bone mineral density compared to isogenic heterozygous control offspring from *Eed*^*wt/del*^ growing oocytes. Our findings are reminiscent of weight gains observed in offspring in other epigenetic models, including mice with disrupted maternal imprinting of H19 [39]. This is an interesting parallel considering recent evidence has demonstrated a role for H3K27me3 in DNA methylation-independent maternal imprinting [34].

Growth trajectories were similar between offspring from all *Eed* oocyte genotypes and mice from *Eed*^*del/del*^ growing oocytes remained moderately larger throughout adulthood (18 weeks of age), although this overgrowth phenotype was ameliorated through time. These phenotypic effects demonstrate the importance of maternal PRC2 for establishing fetal growth patterns and provide evidence that maternal EED regulates offspring growth and postnatal outcomes. Linear regression analysis indicated that litter size did not strongly correlate with weight at PND2. Therefore, while litter size may make a minor confounding contribution to PND2 offspring size, the majority of the overgrowth phenotype was attributable to differences in EED-dependent regulation in the oocyte.

In contrast to the offspring overgrowth observed for loss of EED in the oocyte, we found that oocytes lacking EZH2 resulted in reduced offspring birth weight. The observed EZH2-dependent growth restriction was consistent with a previous study [15]. However, the pattern of inheritance for EED-mediated effects to offspring was not consistent with *Ezh2* regulating a clear epigenetic inheritance effect from the oocyte as HET offspring generated from *Ezh2* ^*wt/del*^ and *Ezh2* ^*del/del*^ oocytes were equivalent, but WT and HET offspring differed in birth weight. In contrast, loss of *Eed* in oocytes resulted in postnatal overgrowth in HET offspring generated from *Eed* ^*del/del*^ oocytes compared to HET offspring from *Eed* ^*wt/del*^ oocytes. As these offspring were isogenic and derived from oocytes that lacked EED during oocyte growth and preimplantation development, it appears that these effects are mediated by altered epigenetic patterning in the oocyte and early life. Furthermore, this *Eed-*mediated, inherited phenotype persisted into the later stages of life (> 18 weeks old). Moreover, while in the previous study, the EZH2-mediated growth restriction was resolved by 4 weeks of age [15], the increased offspring size initiated by lack of EED in the oocyte was more persistent and affected animals through adulthood.

Despite the dependence of PRC2 on EZH2 and EED for catalysing H3K27me3, offspring generated from *Eed*^*del/del*^ and *Ezh2*^*del/del*^ growing oocytes produced remarkably different overgrowth and growth restriction phenotypes in offspring. Growth restricted offspring were produced from *Ezh2*^*del/del*^ oocytes, but there was no difference in weight between HET and HET offspring from *Ezh2*^*del/wt*^ and *Ezh2*^*del/del*^ oocytes, or between WT and WT offspring from *Ezh2*^*del/wt*^ and *Ezh2*^*wt/wt*^ oocytes. However, there was a significant difference in weight between genetically different HET and WT offspring. These observations indicate that the growth restriction observed in *Ezh2* HET vs WT offspring is dependent on *Ezh2* heterozygosity rather than on epigenetic or maternal factor differences identified for EED. One possible explanation is that haploinsufficiency of PRC2 occurs due to compensation for the loss of EZH2 by EZH1 in *Ezh2*^*del/del*^ growing oocytes, whereas PRC2 function is entirely lost in *Eed*^*del/del*^ oocytes. However, this scenario seems unlikely as H3K27me3 levels were similar in *Eed*^*del/del*^ and *Ezh2*^*del/del*^ oocytes, but offspring phenotypes differed substantially for these two models. Moreover, although growth restriction was related to *Ezh2* heterozygosity in offspring, *Eed* heterozygosity did not result in a growth phenotype.

An alternative explanation is that the functions of EZH2 and EED differ in these models. EED is known to function only in PRC2 and is essential for PRC2 function [40]. However, EZH2 can methylate non-histone protein targets [41–43] and result in phenotypic change that is PRC2 independent. For example, EZH2 methylates PLZF in T cells, leading to the ubiquitination of PLZF and its subsequent degradation [43]. Furthermore, T cell-specific deletion of *Ezh2* did not affect H3K27me3 levels and induced rapid expansion of natural killer T (NKT) cells. In contrast, conditional deletion of *Suz12* or *Eed* halted NKT cell development due to PRC2 destabilisation [43]. Furthermore, PLZF exerts growth-suppressive activities [44] and regulates limb-axial skeletal patterning in mice [45]. Interestingly, skeletal development is compromised when *Ezh2* is deleted from mesenchymal stem cells in mice, resulting in reduced skeletal size and reduced body weight [46]. Therefore, although further analyses are required to determine the role of *Ezh2* in this system, it remains possible that the growth restriction observed in *Ezh2* heterozygotes derived from *Ezh2*^*del/del*^ oocytes may result from PRC2 independent EZH2 effects in the embryo rather than reduced H3K27me3 in the oocyte.

Significantly, de novo germline mutations in *EED* or *EZH2* in humans lead to Cohen-Gibson and Weaver syndromes, characterised by overgrowth, skeletal defects and learning/cognitive disabilities [16–18, 21, 22]. As deletion of *Eed* in the maternal germline also resulted in offspring overgrowth, it appears likely that this mouse model, at least in part, reflects the developmental defects typified in Cohen-Gibson syndrome patients. In mice, this appears to be either an epigenetically inherited phenomenon, or to result from loss of a maternal factor activity of EED, for which the only known activity is to regulate epigenetic state through H3K27 methylation. De novo germline mutations in human *EED* lead to Cohen-Gibson, also characterised by fetal overgrowth. Moreover, EED is very highly conserved between mice and humans and loss of EED in the oocyte results in similar offspring phenotypes in both species. Therefore, the *EedZP3Cre* mouse model appears highly relevant for both the study of maternal epigenetic inheritance and Cohen-Gibson syndrome in humans.

An apparent anomaly in the mouse and human models is that murine offspring produced from oocytes lacking *Ezh2* were born with growth restriction, but patients with de novo missense mutations in either *EZH2* or *EED* were born with overgrowth [16–18]. The sequencing data presented here demonstrated that in two affected families, de novo p.Pro132Ser (c.394 C>T) and p.Arg684Cys (c.2050 C>T) changes in *EZH2* were maternally inherited. Both patients presented with increased length at birth (94th and 95th percentiles) and the patient with the p.Arg684Cys (c.2050 C>T) mutation was in the 95th percentile for weight and was delivered preterm. Consistent with the mouse model, which lacks the EZH2 SET domain and is catalytically inactive [47], the maternally derived *EZH2* mutations in both patients reduced histone methyltransferase activity, although the impact of Pro132Ser was less severe than that mediated by Arg684Cys [23]. The reasons for the differing phenotypes in mouse and human (i.e., growth restriction in mouse and overgrowth in Weaver patients) are not clear. However, mesenchymal stem cell-specific *Ezh2* deletion in mice resulted in large offspring with increased size and weight in heterozygous animals, but reduced size and weight in homozygous animals, demonstrating that partial loss of *Ezh2* activity (via heterozygosity of a loss-of-function mutation) and complete loss of *Ezh2* (via deletion) have differing impacts on skeletal size and birth weight [46]. Whether similar effects of EZH2 dosage in human patients result in variable skeletal development remains to be determined. However, human data in DECIPHER associates copy number variations in either EZH2 or EED suggesting that both over- and under-growth can be related to alterations of PRC2 dosage [48].

Epigenetic modifications are established and removed through the activity of specific chromatin modifying complexes, which can be altered by mutations, dysregulated transcription, drugs or other environmental influences. The most prominent examples of epigenetic dysregulation have been found in cancer, with mutations in human tumours commonly detected in genes that regulate chromatin organisation [49]. For example, gain-of-function mutations in *EZH2*, *EED* or *SUZ12* occur in multiple cancer types, and both EZH2 and EED are current targets for cancer therapy [49]. A prominent example is provided by Tazemetostat (Epizyme, Inc.), an EZH1/2 inhibitor in stage I/II clinical trials for treatment of refractory malignant mesothelioma, lymphomas and a number of other tumours [50]. Significantly, as observed in *Eed* and *Ezh2* deleted oocytes, H3K27me3 is substantially and rapidly reduced in growing oocytes of mice treated with pre-clinical doses of Tazemetostat for just 10 days [51]. While the impacts of maternal Tazemetostat treatment on offspring outcomes remain unknown, our study demonstrates that loss of H3K27me3 in growing oocytes substantially affects life-long offspring outcomes.

Importantly, a range of cancers affect men and women of reproductive age, including tumours carrying gain-of-function mutations in *EZH2*. Our mouse model provides evidence that loss of maternal PRC2 function in the germline of adult females disrupts H3K27me3 and results in defects in offspring growth. Based on these findings, it is likely that drugs targeting PRC2 will alter the epigenome in the oocytes of patients undergoing cancer therapy, raising the possibility that these drugs will affect health outcomes in offspring should the patient conceive during or soon after treatment [52]. Indeed, since the oocyte pool in all women is finite, and oocyte growth and maturation spans 12 months in humans, these drugs may have effects in patients’ oocytes long after the termination of treatment. Further evaluation of the impacts of drugs that target PRC2 and other epigenetic modifier enzymes on oocytes and sperm is essential to assess the risks of these drugs in reproductive biology. Such studies would reveal the potential risks of drugs that target epigenetic modifier complexes and would facilitate development of more informed clinical approaches for patients of reproductive age [52], potentially including germline preservation or avoidance of pregnancy after treatment has been completed.

## Conclusions

Using a genetic approach that deleted *Eed* specifically in the growing oocyte, we have demonstrated that this conserved and developmentally important epigenetic modifier mediates programming effects in the oocyte and in the earliest stages of development that are important for life-long outcomes in mice. Further understanding of this and other similar models is essential for determining how epigenetic modifiers regulate early life and long-term developmental outcomes in human health and the developmental origins of disease.

## Methods

### Mouse strains, animal housing, breeding and ethics

Mice were housed at Monash Medical Centre Animal Facility using a 12-h light-dark cycle. Food and water were available ad libitum and room temperature was 21–23 °C with controlled humidity. With the exception of breeding pairs and newborn mice (up to 3 weeks old), males and females were weaned at 21 days and kept in cages of up to five individuals. All animal work was undertaken in accordance with Monash University Animal Ethics Committee (AEC) approvals. *Zp3Cre* mice (C57BL/6-Tg 93knw/J; Jackson Labs line 003651) were constructed by Professor Barbara Knowles and obtained from The Jackson Laboratory. *Eed* floxed mice (*Eed*^*fl/fl*^*)* (B6; 129S1-*Eed*^*tm1Sho*^/J; Jackson Labs line 0022727) were constructed by Stuart Orkin and colleagues [36] and obtained from the Jackson Laboratory. *Ezh2* floxed mice (*Ezh2*^*fl/fl*^*)* were constructed by Alexander Tarakhovsy and colleagues [47]. The *Eed* and *Ezh2* lines were backcrossed to a pure C57BL6/J and shared with us by Rhys Allen and Marnie Blewitt, Walter and Eliza Hall Institute for Medical Research, Melbourne.

### Genotyping

Colony maintenance animals were genotyped via tail collection at PND2 or ear punch at weaning by Transnetyx (Cordova, TN) using real-time PCR assays designed for each gene (details available on request). Assays were designed based on the genomic structure of *Eed* and *Ezh2* in relation to the conditional genetic modifications established in [36] and [47], respectively.

### Tissue fixation and embedding

Ovaries were fixed in 4% paraformaldehyde (PFA) in PBS overnight at 4 °C. Samples were then washed in PBS and left in a 30% sucrose solution overnight at 4 °C. Samples were then placed in disposable cryostat moulds (Sakura Finetek, #4565) filled with OCT (Sakura Finetek, #4583) and frozen in dry ice. Blocks were stored at − 80 °C.

### Immunofluorescence

Eight micron sections were cut from OCT embedded ovaries fixed in 4% PFA, mounted on Superfrost Plus slides and dried for 5 min before immersing in 1 × PBS. Sections were then permeabilised by incubation in 1% Triton × 100 (Sigma, #T8787) in PBS for 10 min at room temperature (RT). Slides were washed in PBS. Sections were blocked in PBS containing 5% BSA (Sigma, #A9647) and 10% donkey serum (Sigma, #D9663) and incubated for 45 min at RT. Blocking solution was replaced by PBS containing 1% BSA and appropriately diluted H3K27me3 antibody (1:400, rabbit anti-H3K27me3 Cell Signaling Technologies #C36B11) and incubated for 1 h at RT. Slides were washed three times for 5 min in PBS and secondary antibodies diluted in 1% BSA in 1 × PBS according to antibody dilutions (1:300, donkey anti-rabbit 594, Alexa Fluor Life Technologies #A21207. Secondary antibody incubation was carried out in a dark box for 1 h at RT. Slides were washed three times in PBS (5 min each wash) and mounted in ProLong Gold**®** containing DAPI (Life Technologies, #P36931) and left in a dark box overnight to dry. For control slides, only a secondary antibody was applied. Confocal images were taken as single optical sections using a Nikon**®** C1 inverted Confocal microscope. All pictures were taken at × 80, using a × 40 oil immersion lens.

### Phenotypic analysis of offspring

Offspring were weighed at PND2 and/or 30, 49 and 130. At PND30, 49 and 130, female and male mice were analysed separately to account for age-related sex-specific differences. Crown to rump (rump defined by base of tail) and nose to rump measurements were taken by one individual to minimise variances in method. Maternal genotypes were concealed to ensure no bias during collection of data.

### Lunar PIXImus DEXA (bone mineral density, lean muscle, fat content)

PND2 offspring were measured, euthanised and stored at − 20 **°**C. Lunar PIXImus for small animals was used to measure bone and tissue composition using dual-energy X-ray absorptiometry (DEXA). Quality control is ensured by calibrating Lunar PIXImus to a QC phantom upon each run. Animal is place inside the region of interest (ROI), and scan is completed, *n* = 61. All data was collected using Lunar Piximus 2.0 software.

### Long-range PCR and sequencing of patient and parental DNA samples

Regions spanning the defined mutations in the EZH2 gene of patients were amplified using long-range PCR in patient and parental DNA samples and processed for single molecule real-time (SMRT) sequencing (Pacific Bioscience) by the Leiden Genome Technology Centre, Department of Human Genetics, Leiden University Medical Center, the Netherlands. All samples were obtained by Professor William Gibson under University of British Columbia and British Columbia Children’s Hospital Human Ethics approval numbers H08-00784, H09-01228 and H10-03215, University of British Columbia, Vancouver, Canada. Sample preparation and workflow was carried out as previously described [53]. Primer sequences are included in Additional file 1.

### Statistical analysis

One-way ANOVA plus Tukey’s post hoc test was used to statistically analyse all quantitative data. Pearson *r* correlation test used to analyse weight vs. litter size. PRISM software v6.0e; GraphPad Prism 7 was used to analyse and graph data sets. Where data are presented graphically, statistically significant (*P* < 0.05) post hoc outcomes are represented by an asterisk.

A linear mixed model with litter ID as a random effect was used to test for differences among offspring groups while accounting for the relationship between offspring weight and litter size. Tests of significance for fixed effects were undertaken using Satterthwaite approximation for degrees of freedom and type III sums of squares for ANOVA. Linear mixed models were implemented in R v3.3.2 (R Core [54]) using lme4 v1.1-13 and lmerTest v2.0-33 [55, 56].

## Additional file

Additional file 1: Supplementary information. (PDF 389 kb)

## Abbreviations

E: Embryonic day
EED: Embryonic Ectoderm Development
eGFP: Enhanced GFP
EZH1: Enhancer of Zeste 1
EZH2: Enhancer of Zeste 2
H3K27me3: Trimethylated lysine 27 on histone 3
MVH: Mouse vasa homologue (also known as DDX4)
*Nsd*: No significant difference
PRC2: Polycomb repressive complex 2
SUZ12: Supressor of Zeste 12
Zp3-Cre: Zona pellucida 3 Cre recombinase
