# Supplementary material for: Loss of maternal EED results in postnatal overgrowth
Source: Clin Epigenetics. 2018 Jul 13;10:95. doi: 10.1186/s13148-018-0526-8 (PMC6045828; doi:10.1186/s13148-018-0526-8)
Supplement: Supplementary file 1 — Supplementary information. (PDF 389 kb) [file 13148_2018_526_MOESM1_ESM.pdf]

Supplementary information:

Loss of maternal EED results in postnatal overgrowth

Lexie Prokopuk[1], Jessica M. Stringer[1,2], Craig R. White[3], Rolf H.A.M Vossen[4], Stefan J. White[4], Ana S.A. Cohen[5], William T. Gibson[5] and Patrick S. Western[1#]

[1]Centre for Genetic Diseases and Centre Reproductive Health, Hudson Institute of Medical Research and Department of Molecular and Translational Science, Monash University, Clayton, Victoria, Australia 3168.

[2]Monash Biomedicine Discovery Institute, Monash University, Clayton, Victoria, Australia 3800.

[3]Centre for Geometric Biology, School of Biological Sciences, Monash University, Clayton, Victoria, Australia 3800.

[4]Leiden Genome Technology Centre, Department of Human Genetics, Leiden University Medical Center, Leiden, the Netherlands

[5]Dept. of Medical Genetics, University of British Columbia and British Columbia Children's Hospital Research Institute, Vancouver, BC, Canada

[#]Corresponding author

patrick.western@hudson.org.au

Tel: +61 3 8572 2673

Supp Fig 1.

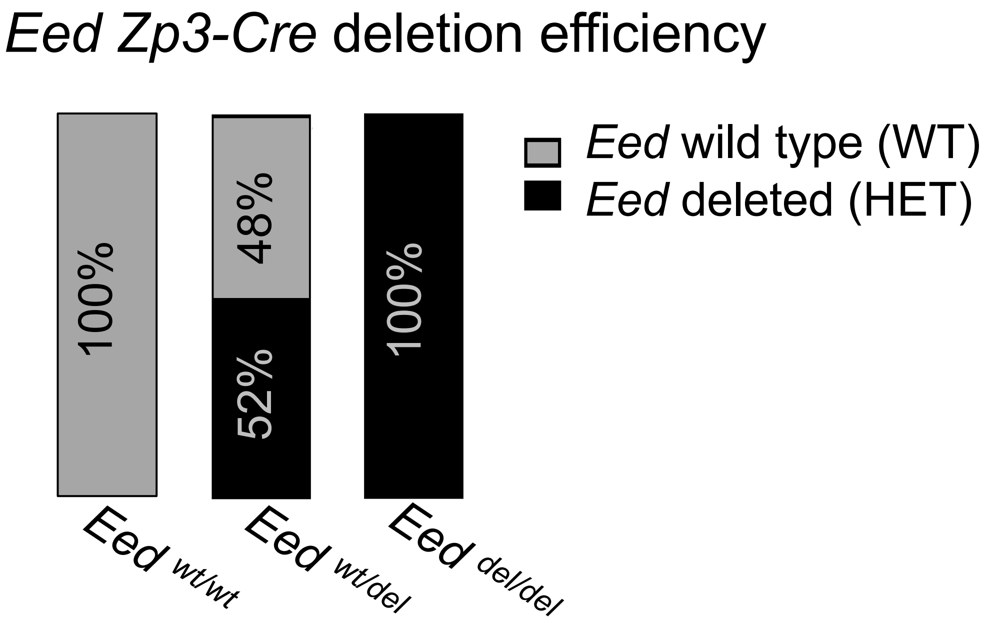

Eed Zp3-Cre deletion efficiency
100%
48%
52%
100%
Eed wild type (WT)
Eed deleted (HET)
Eed wt/wt
Eed wt/del
Eed del/del

Supp Fig 1. Deletion efficiency of *Eed;Zp3-Cre* transgene: Genotyped offspring from *Eed* floxed females (*Eed*$^{fl/fl}$, *Eed*$^{wt/fl}$*;Zp3*-Cre and *Eed*$^{fl/fl}$*;Zp3*-Cre) mated to wild type males. Grey bars represent genotyped WT offspring from *Eed*$^{wt/wt}$ or *Eed*$^{wt/del}$ growing oocytes; black bars represent genotyped HET offspring from *Eed*$^{wt/del}$ or *Eed*$^{del/del}$ growing oocytes (*Eed*$^{fl/fl}$ n= 54, *Eed*$^{wt/fl}$*;Zp3*-Cre n=134 (wt=64, del=70) , *Eed*$^{fl/fl}$*;Zp3*-Cre n=33; *Chi-square test, nsd).*

Supp Fig 2.

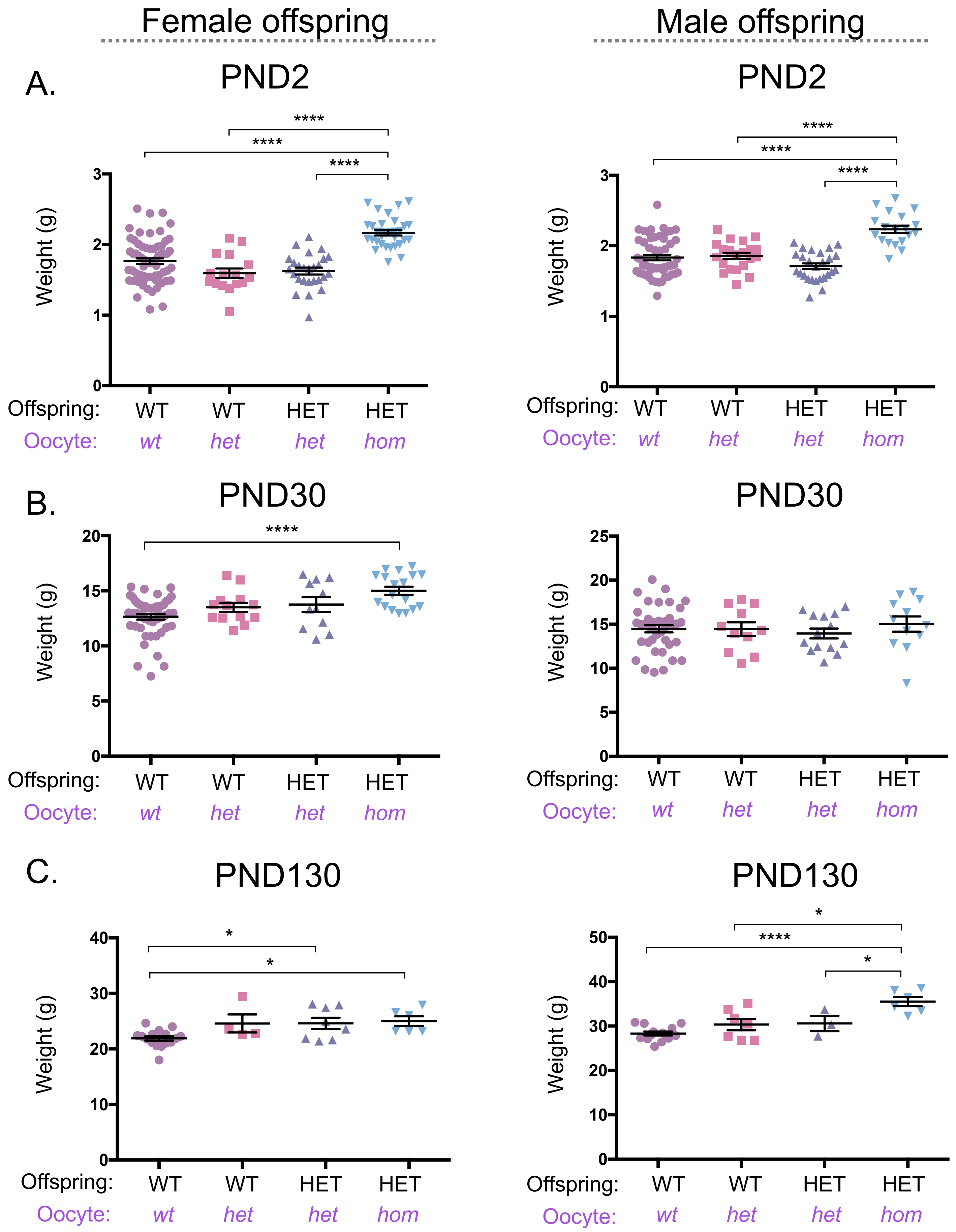

Female offspring
Male offspring
A.
PND2
PND2
Weight (g)
****
****
****
Offspring: WT WT HET HET
Oocyte: wt het het hom
B.
PND30
PND30
C.
PND130
PND130
*

Supp Fig 2. Increased weight of offspring from *Eed*$^{del/del}$ oocytes showed no sex-bias and was ameliorated over time: Weights of offspring at 2, 30 and 130 days old. Female (left graph) and male (right graph) offspring from wild type fathers mated to *Eed*$^{fl/fl}$, *Eed*$^{fl/del}$*;Zp3*-Cre and *Eed*$^{fl/del}$*;Zp3*-Cre mothers. Growing oocyte genotype represented in purple: *Eed*$^{wt/wt}$ *(wild type, wt), Eed*$^{wt/del}$*(wt or heterozygous, het)* and *Eed*$^{del/del}$ *(homozygous, hom).* Offspring genotype represented in black: *Eed* WT offspring from *Eed*$^{wt/wt}$ growing oocytes; *Eed* WT offspring from *Eed*$^{wt/del}$ growing oocytes; *Eed* HET offspring from *Eed*$^{wt/del}$ growing oocytes and *Eed* HET offspring from *Eed*$^{del/del}$ growing oocytes. **A.** Female PND2 offspring: *Eed*$^{wt/wt}$ n= 63, *Eed*$^{wt/del}$ n=68, *Eed*$^{del/del}$ n=32; Male PND2 offspring: *Eed*$^{wt/wt}$ n= 53, *Eed*$^{wt/del}$ n=74, *Eed*$^{del/del}$ n=19. **B.** Female PND30 offspring: *Eed*$^{wt/wt}$ n= 49, *Eed*$^{wt/del}$n=24, *Eed*$^{del/del}$ n=18; Male PND30 offspring; *Eed*$^{wt/wt}$ n=40, *Eed*$^{wt/del}$n=25, *Eed*$^{del/del}$ n=12. **C.** Female PND130 (18 week old) offspring: *Eed*$^{wt/wt}$ n= 16, *Eed*$^{wt/del}$n=12, *Eed*$^{del/del}$ n=6; Male PND130 (18 week old) offspring; *Eed*$^{wt/wt}$ n=14, *Eed*$^{wt/del}$n=10, *Eed*$^{del/del}$ n=6. ****P<0.0001, One-way ANOVA plus post-hoc Tukey's multiple comparisons test. Error bars +/- SEM.

Supp Fig 3.

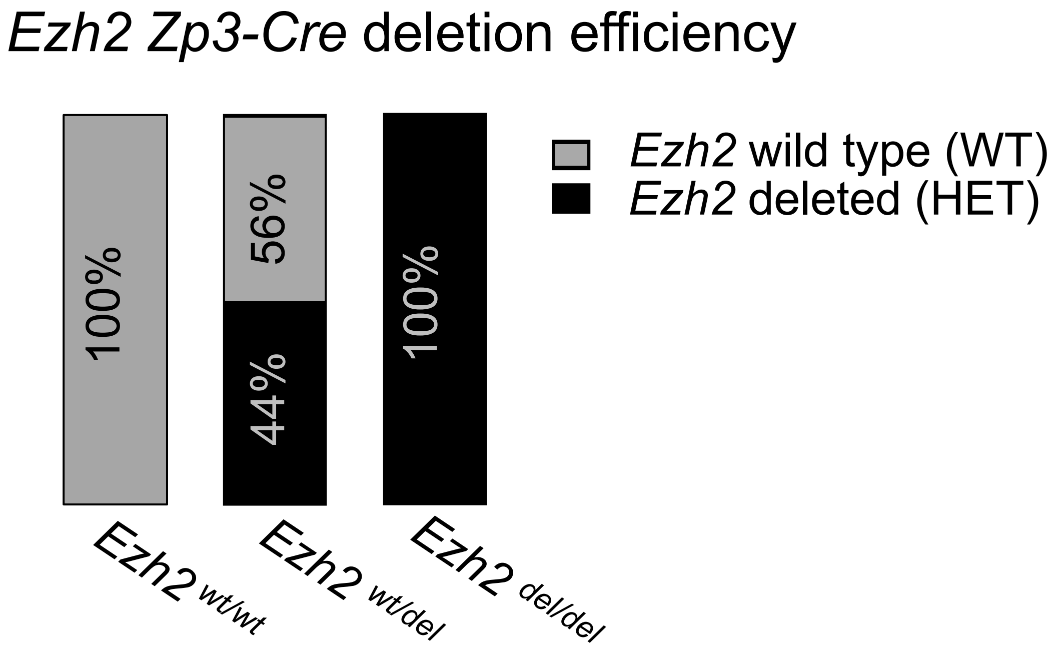

Ezh2 Zp3-Cre deletion efficiency
100%
56%
44%
100%
Ezh2 wild type (WT)
Ezh2 deleted (HET)
Ezh2 wt/wt
Ezh2 wt/del
Ezh2 del/del

Supp Fig 3. Deletion efficiency of *Ezh2;Zp3-Cre* transgene: Genotyped offspring from *Eed* floxed females (*Ezh2*$^{fl/fl}$*, Ezh2*$^{wt/fl}$*;Zp3*-Cre and *Ezh2*$^{fl/fl}$ *;Zp3*-Cre) mated to wild type males. Grey bars represent genotyped WT offspring from *Ezh2*$^{wt/wt}$ or *Ezh2*$^{wt/del}$ growing oocytes; black bars represent genotyped HET offspring from *Ezh2*$^{wt/del}$ or *Ezh2*$^{del/del}$ growing oocytes. (*Ezh2*$^{fl/fl}$ n=23*, Ezh2*$^{wt/fl}$*;Zp3*-Cre n, wt=35 and del=28 and *Ezh2*$^{fl/fl}$ *;Zp3*-Cre n=19) *Chi-square test, nsd).*

Supplementary methods:

Primer sequences of long range sequencing of patient and parental DNA samples:

Fragment	M13-F primer	M13-R primer
EZH2-1	TGTAAAACGACGGCCAGTAGCGTGTTCTTACCTGTGGG	CAGGAAACAGCTATGACCGGACCTTTCAGCGACTCCAA
EZH2-2	TGTAAAACGACGGCCAGTTGCATAGCCTGTGCTGTAGG	CAGGAAACAGCTATGACCGGCAAACACCACAAGCTAGG
EZH2-3	TGTAAAACGACGGCCAGTGAGTTAACTCTATCAGCCAGG	CAGGAAACAGCTATGACCAGCAGATGTCAAGGGATTTCC
